# Intravital microscopy for nanomedicine: investigating nanoparticle–tissue interactions in the native state

**DOI:** 10.1039/d5ra08785f

**Published:** 2026-01-26

**Authors:** Giovanni M. Saladino

**Affiliations:** a Department of Radiology, School of Medicine, Stanford University Stanford CA 94305 USA gmsaladino@stanford.edu

## Abstract

Nanomedicine is an evolving field that employs nanoparticles (NPs) for drug delivery systems, molecular imaging, and therapeutic interventions. However, characterizing NP–cell interactions poses significant challenges, mainly due to the limitations of traditional postmortem histological methods, such as immunofluorescence staining. These techniques yield a single time-point analysis and include lengthy preprocessing steps that can induce artifacts and alter tissue architecture. They result in the clearance of blood components, preventing the evaluation of circulating NPs. Moreover, the rapid sequestration of NPs by organs, particularly the liver and spleen, limits the understanding of their biodistribution and pharmacokinetic profiles. In this context, intravital microscopy (IVM) has emerged as a unique imaging technology that allows real-time visualization of NP behavior within living organisms. By maintaining the physiological context, IVM enables the investigation of NP circulation, tissue accumulation, and cellular interactions at the microscopic scale. This review focuses on the recent uses of IVM to investigate NP–cell interactions in nanomedicine, highlighting its impact on advancing knowledge of NP dynamics, uptake, and the development of more targeted NP-based therapies.

## Abstract 2

This review provides an overview of surgical procedures, nanoparticle design considerations, and imaging strategies for intravital microscopy in nanomedicine.

## Graphical abstract

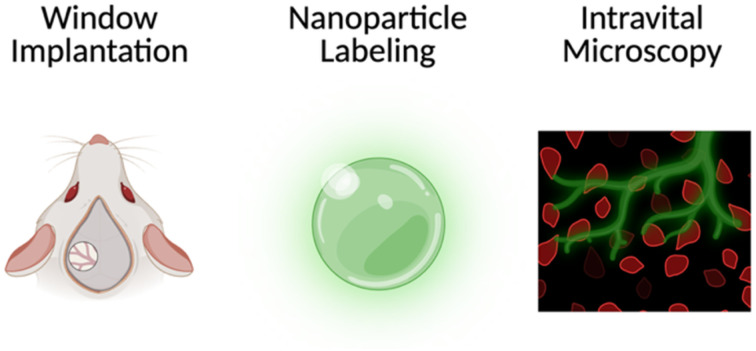

## Background

Nanomedicine is rapidly emerging as a transformative field within healthcare, employing the unique properties of nanomaterials to enhance disease diagnosis, treatment, and prevention.^1–5^ By enabling targeted drug delivery, improved diagnostic imaging, and novel therapeutic approaches, nanomedicine holds the promise of revolutionizing patient care and addressing complex medical challenges more effectively than traditional methods.^6,7^ However, despite its potential, the interactions between nanoparticles (NPs) and cells *in vivo* remain incompletely understood. This gap in knowledge poses significant challenges, as the efficacy and safety of nanomedicine applications are heavily dependent on these interactions. Continued research is essential to study the underlying mechanisms of NP–cell dynamics, providing key information leading to improved NP design and strategies for clinical use.

**Fig. 1:**
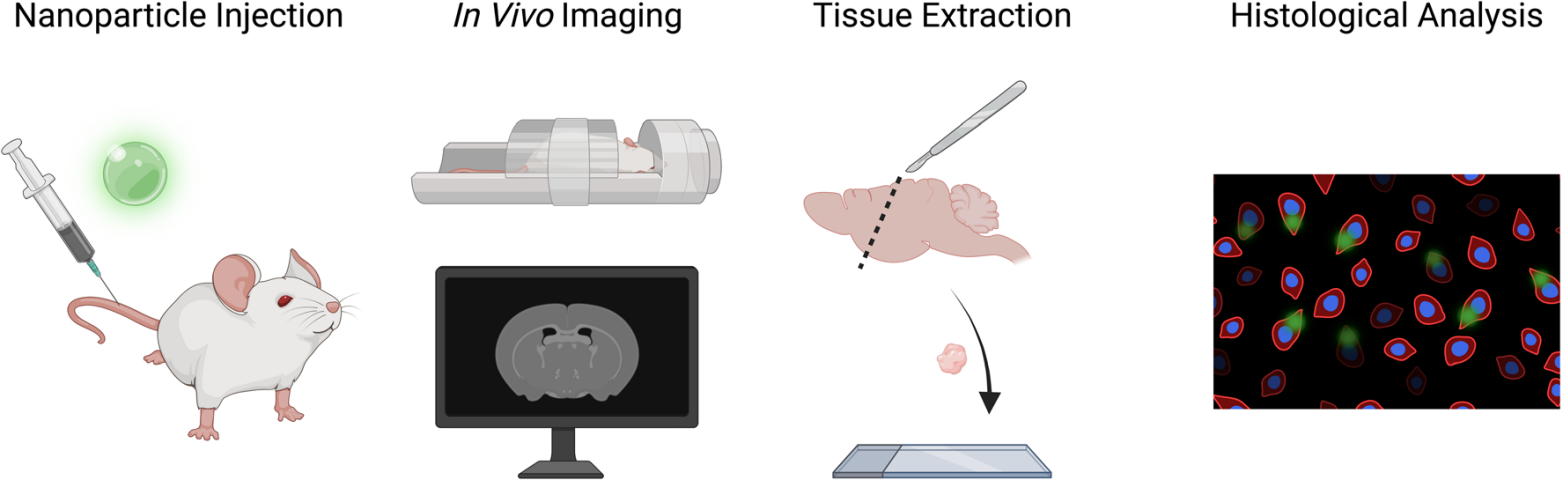
Schematic representation of the conventional steps for *in vivo* studies in mice with dual-mode NPs. After NP injection, macroscopic imaging (*e.g.*, MRI) is performed. Subsequently, mice are euthanized at a fixed time point and their tissue is extracted for postmortem histological analysis. NP detection with optical microscopy is enabled by conjugation with specific fluorophores or intrinsic fluorescence properties.

Typically, postmortem histology, including techniques like immunofluorescence, offers valuable insights into cellular and tissue characteristics at a specific point in time, providing a microscopic analysis of biological processes following death (Fig. 1).^8–11^ However, this approach has inherent limitations that can hinder a comprehensive understanding of dynamic *in vivo* interactions. The constraint of a single time-point means that any temporal variations in cellular behavior or disease progression are lost, potentially neglecting critical information. Furthermore, numerous preparation steps are required for immunofluorescence staining, including tissue collection, fixation in a fixative solution (*e.g.*, formaldehyde), embedding in paraffin, sectioning into thin slices, deparaffinization, antigen retrieval, blocking to minimize non-specific binding, incubation with primary antibodies, washing to remove unbound antibodies, applying fluorescently labelled secondary antibodies, further washing, and finally mounting for imaging. This process can alter the tissue's original state, leading to artifacts that misrepresent the actual biological conditions prior to death.^12^ Additionally, the histological analysis process often requires several days, thus slowing down the data collection and reducing efficiency in assessing the treatment and diagnostic potentials of the tested NPs. These factors evidence the need for complementary *in vivo* techniques that can enable the investigation of NP–cell interactions in real-time, enhancing our understanding of nanomedicine while avoiding the disadvantages of traditional postmortem analysis.

Moreover, a significant limitation of immunofluorescence in tissue analysis is its inability to effectively analyze NPs in circulation. During the preparation process for histological studies, blood vessels are cleared of blood during the fixation and embedding of tissues. This clearance not only removes the NPs from circulation but also impedes the observation of their interactions with vascular structures. As a result, crucial information regarding the behavior and distribution of NPs within the bloodstream and their potential therapeutic effects is lost. Analyzing circulating NPs is fundamental in the study of nanomedicines because their biodistribution and clearance from the bloodstream can significantly influence their therapeutic efficacy and safety.^13–15^ Once administered into the body, NPs are typically uptaken by organs such as the liver and spleen due to several factors including size, shape, surface charge, and NP coating, which affect their interactions with cells and proteins in the blood.^16–18^ Understanding the kinetics of NP circulation becomes fundamental for optimizing NP design and improving targeting capabilities. This point highlights the necessity of developing *in vivo* imaging techniques for real-time monitoring of NPs as they circulate through the body, allowing researchers to gain insights into *in vivo* NP biodistribution, pharmacokinetics, and interactions with target tissues.

Intravital microscopy (IVM) is an imaging technique for the real-time observation of biological processes within living organisms,^19,20^ thus providing an opportunity to study NPs in their native state. This tool permits to visualize tissues and monitor the dynamics of circulating NPs as they interact with various organs and cells in real time. By preserving the physiological context often lost in traditional histological techniques, IVM can capture dynamic processes such as NP circulation and uptake, as well as their interactions with immune cells and vascular structures. This property can promote key findings about NP distribution, mechanisms of action, and targeting efficacy, while also allowing the exploration of how factors like inflammation or disease states influence NP behavior.

Finally, the application of IVM could significantly expand our understanding of circulating NPs *in vivo*, promoting innovations in nanomedicine and the development of more effective and safer NP-based therapies.

**Fig. 2:**
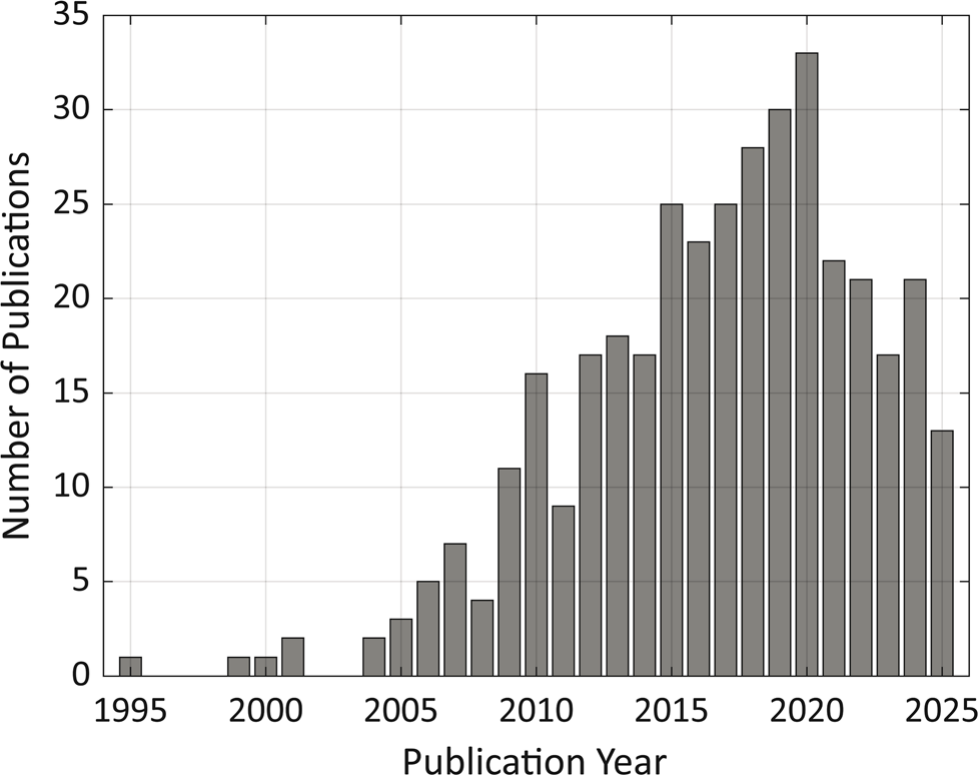
Bar plot showing the number of PubMed-indexed articles per year returned by the query “Nanoparticle Intravital” (all fields). The search was performed on 23 October 2025.

The bar chart illustrating the number of articles published and indexed in PubMed focusing on intravital imaging of NPs (Fig. 2) clearly depicts an upward trend in the volume of research output over the last decade. This increase is indicative of the growing interest and recognition of the importance of intravital imaging techniques in understanding the NP fate *in vivo*. This trend evidences the role that IVM is playing in advancing research in nanomedicine.

The present review focused on the recent applications of IVM for exploring NP–cell interactions within the field of nanomedicine. By highlighting the innovative applications of IVM, the aim was to investigate how this technique has advanced our understanding of the biodistribution, cellular uptake, and targeting mechanisms of NPs *in vivo*.

## Surgical procedures in mice

**Fig. 3:**
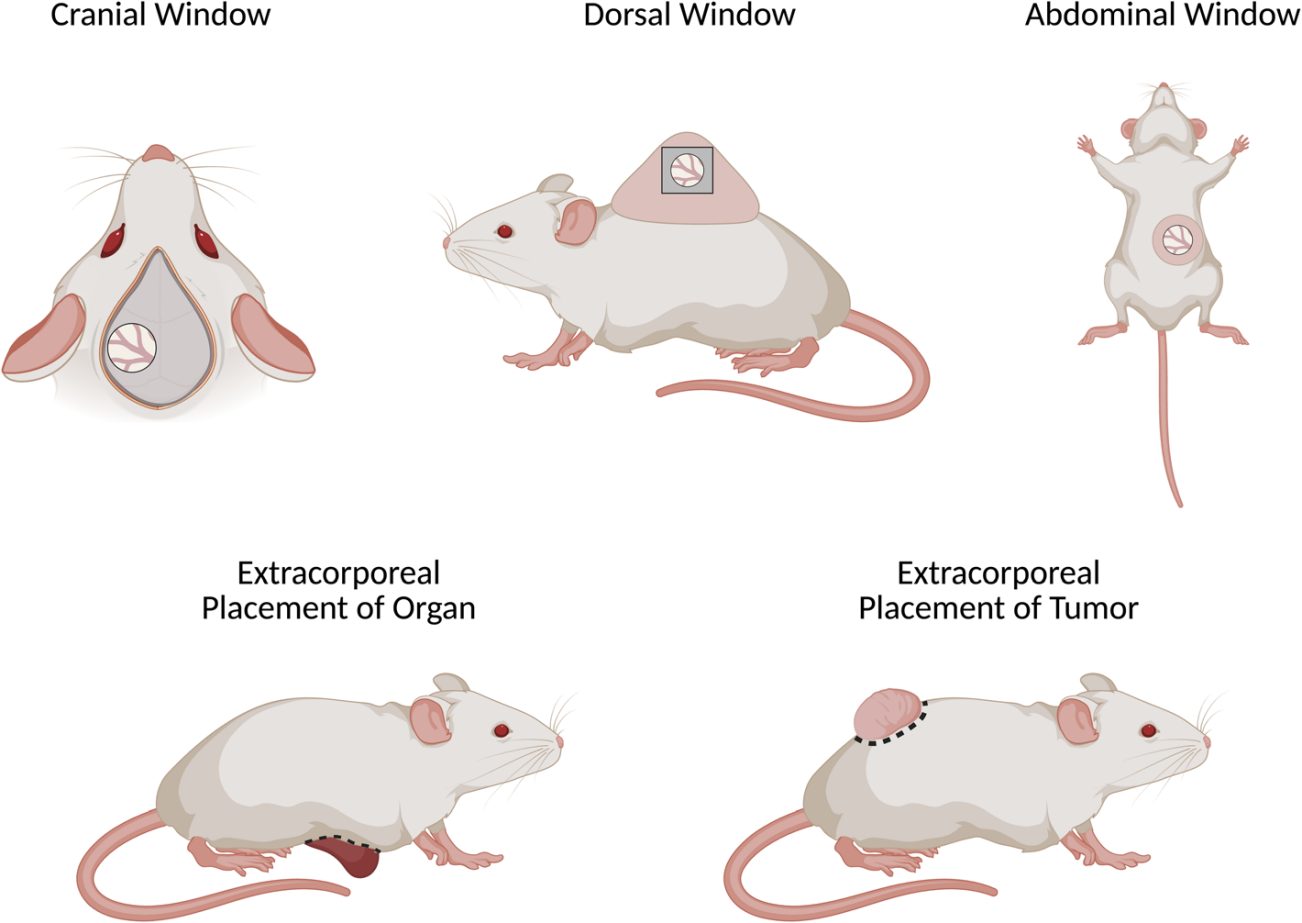
Most common surgical procedures enabling IVM imaging on mouse models, including window implantation (cranial, dorsal, abdominal) and extracorporeal placement.

IVM requires rigorous preparation of animal models to enable real-time imaging of NPs within the physiological context. The preparation process involves several critical steps, including surgical interventions and post-operative care to ensure the well-being of the animals. A critical component of IVM is the implantation of optical windows or the extracorporeal placement of the organ/tumor (Fig. 3). The implanted windows allow for *in vivo* imaging without the need to disrupt the tissue during the experiment and enable longitudinal studies. An incision is made in the skin to expose the underlying tissue and a small section of the tissue/bone.

A biocompatible optical window (*e.g.*, a thin cover glass or transparent polymer disc) is implanted and secured to the tissue using sutures or adhesive materials. The design of the implant must also consider compatibility with other imaging modalities, such as magnetic resonance imaging (MRI), to facilitate potential multimodal imaging applications. The implanted window provides access to the tissue for imaging while maintaining the natural environment and integrity of the vascular and cellular structures. The successful implantation of optical windows and preparation of the animals are crucial for enabling high-resolution imaging of NP–cell interactions in real time using IVM.

Common sites for IVM analysis include the dorsal skin,^21,22^ which provides good accessibility for visualizing skin-associated processes and vascular dynamics; cranial windows in the skull for studying NP behavior in the brain;^23–25^ in the mesentery, which allows observation of gastrointestinal processes and immune cell interactions. IVM can also be performed directly over tumors or within tumor models, facilitating the study of NP delivery and distribution in the tumor microenvironment.^26,27^ Additionally, IVM on regions such as the femoral vein provide opportunities to visualize cardiovascular dynamics, including murine arterial and venous thrombosis.^28^

IVM can be performed on the liver with both surgical implantation of an abdominal imaging window^29,30^ and extracorporeal placement.^31,32^ IVM on kidneys permits to investigate NP clearance.^33^ Finally, transthoracic window implantation enables IVM on mouse pulmonary micro vessels.^34^

Ear vasculature imaging with IVM offers a non-invasive alternative to window implantation and tissue extracorporeal placement. This method allows the observation and tracking of NP circulation in real-time within the vascular network of the ear,^35^ and enables the study of NP delivery to ectopic tumor models.^36^ However, it facilitates studies related to systemic distribution rather than NP localization in specific tissue or organ.

IVM of dynamic organs is challenged by motion from respiration, heartbeat, and peristalsis. To limit artifacts, mechanical stabilization or physiological gating can be employed.^37,38^ Key limitations of the dorsal windows are the restricted tumor thickness, which may prevent visualization of larger or more heterogeneous lesions, and the reduced local temperature compared to the normal body temperature, which leads to a different tumor growth than in the subcutaneous xenograft models.^39^

During surgery, appropriate analgesia and anesthesia protocols should be followed,^40^ strict aseptic techniques applied to prevent infection, and sufficient time allowed for inflammation to resolve (typically 24–48 h) before imaging to minimize confounding effects.

## Comparative overview of intravital microscopy techniques

IVM enables real-time, cellular- to subcellular-resolution visualization of NPs and their interactions within native tissue microenvironments. IVM includes confocal, spinning-disk, two-photon, three-photon, and light-sheet microscopy, each defined by a different balance of depth, resolution, speed and photodamage. Confocal microscopy provides high spatial resolution near the tissue surface (depth: ∼0–100 µm; voxel size: ∼0.2–0.5 µm; frame rate: ∼0.1–1 Hz) but rapidly loses signal and SNR because both excitation and emission scatter, limiting penetration depth *in vivo* and accelerating photobleaching at higher powers.^41^ These constraints motivated nonlinear excitation, where two-photon microscopy confines excitation to the focal volume, improving SNR and enabling stable optical sectioning several hundred micrometers deep (∼200–800 µm) with reduced out-of-focus bleaching.^42–44^ Two-photon IVM is therefore the most widely used compromise for deep-tissue cellular imaging:^45^ although still point-scanned and power-demanding (power at sample: ∼10–100 mW; voxel size: ∼0.3–0.8 µm; frame rate: ∼0.5–10 Hz), it maintains submicron resolution at depth while slowing bleaching and phototoxicity relative to confocal at equivalent penetration. Three-photon excitation uses longer wavelengths and higher-order confinement to reach millimeter-scale depths in scattering organs such as the cortex (depth: ∼500–1500 µm; voxel size: ∼0.5–1.0 µm; frame rate: ∼0.1–1 Hz; power at sample: ∼50–150 mW), but with the disadvantages of slower acquisition, higher pulse energies and increased risk of focal heating.^46,47^ Light-sheet microscopy is the preferred choice for dynamic studies at the surface level, as it minimizes bleaching and enables rapid volumetric imaging (frame rate: ∼1–10 Hz; voxel size: ∼0.5–2 µm) with high SNR, reaching depth up to ∼200 µm and with a power at sample of ∼1–20 mW.^48^

Overall, the nonlinear optical imaging techniques allow for deeper penetration into tissues (up to 1–1.5 mm), significantly reducing photodamage and enabling selective excitation of fluorescent molecules at defined depths. By utilizing longer wavelengths, which are less scattered in biological tissues, IVM platforms can visualize cellular and NP dynamics within living organisms in greater detail. Owing to their nonlinear excitation, which provides intrinsic optical sectioning and deeper tissue penetration, two-photon and three-photon imaging are especially advantageous for observing cellular interactions in complex microenvironments, such as those found in tumors or vascular systems, thereby enhancing the study of NP–cell interactions and their targeting implications.

## Fluorescence nanoparticle labelling

**Fig. 4:**
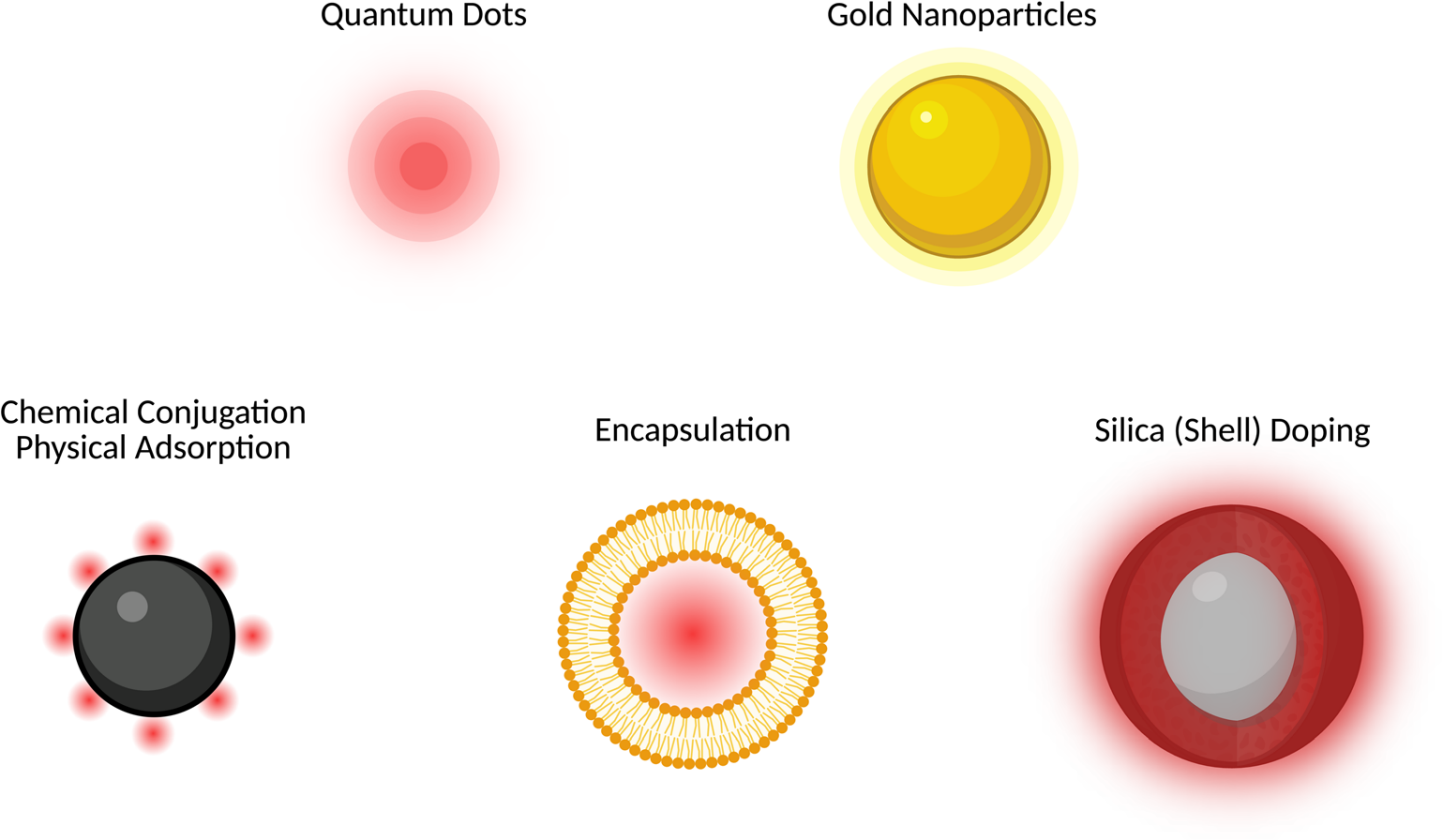
Schematic illustration of fluorescent nanoparticles (NPs) and fluorophore conjugation strategies. Intrinsically fluorescent NPs, such as quantum dots and gold NPs, emit light without external labeling. Fluorophore-labeled NPs are obtained through various methods: (1) chemical conjugation *via* covalent bonds (*e.g.*, amide, thioether) between NP surface groups and dyes or physical adsorption of fluorophores onto the NP surface through non-covalent interactions; (2) encapsulation of fluorophores within liposomes, polymers, or NP matrices; and (3) silica (shell) doping, where dyes are conjugated within a silica core or layer during NP synthesis for enhanced stability and brightness.

Fluorescence NP labelling enables the visualization and tracking of NPs with IVM (Fig. 4). Certain NPs, like quantum dots or gold NPs, have intrinsic photoluminescence properties that allow them to emit light upon excitation.^49–56^ In addition to NPs with intrinsic fluorescence properties, non-fluorescent NPs can be labelled with common fluorophores to enable optical detection. Typical fluorophores used include fluorescein isothiocyanate (FITC), which emits green fluorescence, and cyanine dyes like Cy5, which emit in the far-red spectrum.^57–59^ The labelling process generally involves either physical adsorption or chemisorption of the fluorophores to the NP surface, ensuring that the fluorescent properties are stable and preserved.^60^ Physisorption can result in unstable labeling, causing imaging of free dye instead of the NPs; this can be reduced by using chemisorption or doping strategies, which provide more stable incorporation than physisorption. Fluorescent labels and conjugates can affect the NP charge, protein corona formation, and clearance,^61^ while heavy labeling risks free-label artifacts, so minimal and stable labeling, ratiometric designs, and NIR-II probes should be employed whenever possible.^62^ Furthermore, silica shells and cores have been employed to encapsulate dyes to increase their brightness and extend their photostability.^63–68^ Another common route to achieve optically fluorescent NPs is encapsulating a dye within the core or membrane of a lipidic or polymeric NPs.^69–76^ Finally, label-free imaging techniques, such as Second-Harmonic Generation (SHG), Third-Harmonic Generation (THG), and Coherent Anti-Stokes Raman Scattering (CARS) microscopy, utilize the intrinsic properties of biological tissues and, in specific cases, NPs to detect signals without the need for external fluorescent labels.^77^

## Intravital imaging of nanoparticles

**Fig. 5:**
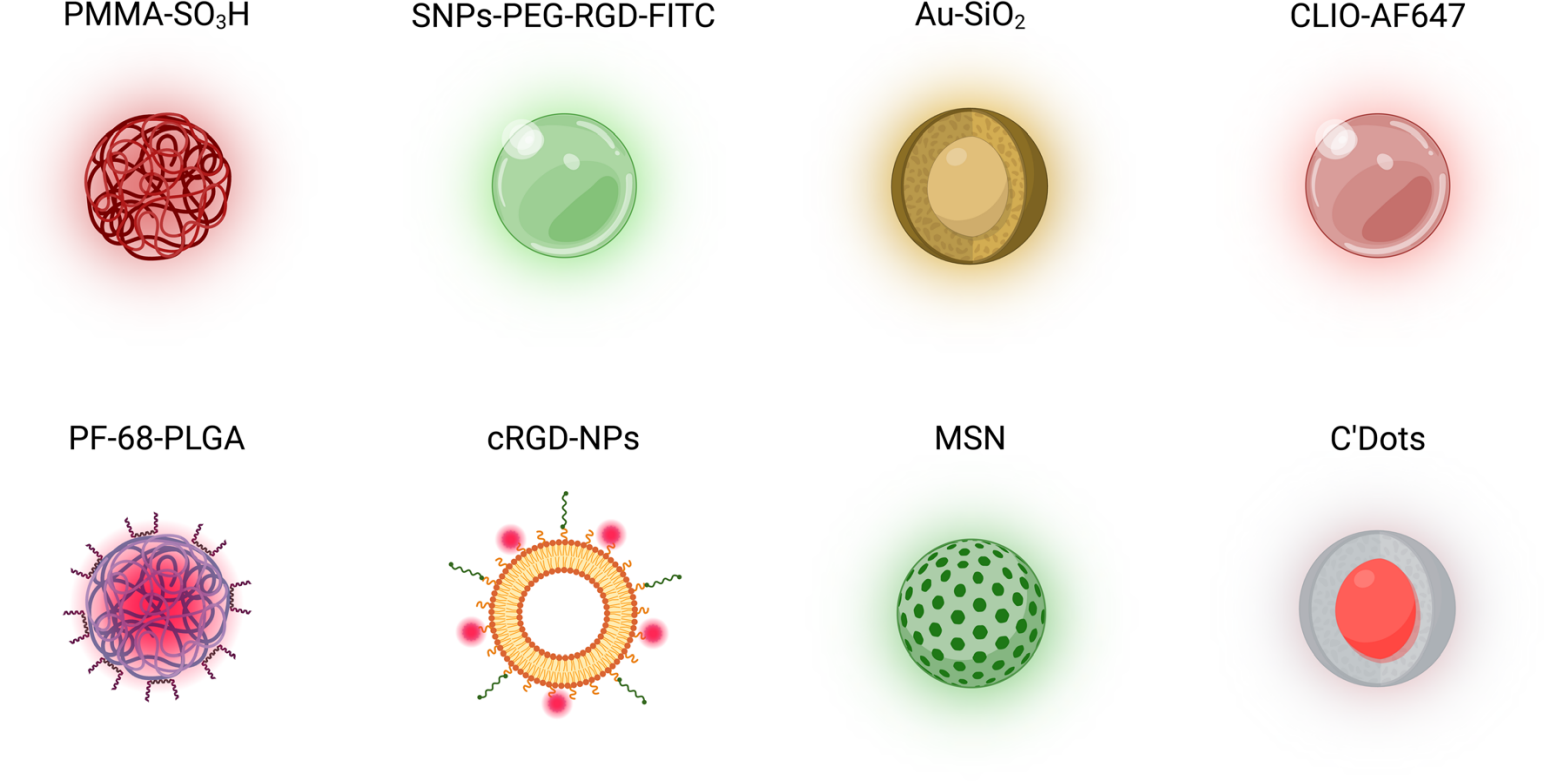
Schematic representation of examples of nanoparticle (NP) designs, including polymeric, lipidic, ceramic, and metallic NPs, employed for *in vivo* studies with IVM. These include poly(methyl methacrylate)-sulfonate (PMMA-SO_3_H) NPs,^25^ PEGylated silicon NPs (SNPs-PEG-RGD-FITC),^79^ core–shell gold–silica (Au–SiO_2_) NPs,^35^ cross-linked iron oxide (CLIO-AF647) NPs,^80^ poly(lactic-*co*-glycolic acid) NPs coated with Pluronic-68 (PF-68-PLGA),^24^ cyclic arginine–glycine–aspartate peptide- (cRGD-) NPs,^81^ mesoporous silica nanoparticles (MSNs),^32^ and Cornell Prime Dots (C'Dots).^82^

Over the past decade, numerous NP designs have been employed to investigate NP–cell interactions utilizing IVM (Table 1, Fig. 5). Choosing bright, photostable fluorescent NPs reduces the need for prolonged illumination, aiming at minimal tissue stress and at operating at safe operational conditions.^78^ An example of designed NPs consists of dye-loaded polymeric NPs engineered for single-particle visualization *in vivo*.^25^ These NPs, composed of hydrophobic poly(methyl methacrylate)-sulfonate (PMMA-SO_3_H) NPs loaded with the dye octadecyl rhodamine B, exhibit enhanced stealth characteristics through adsorption of PEGylated Pluronic F-68 and F-127, conferring resistance to nonspecific protein binding and serum displacement. A significantly higher fluorescence brightness was reported compared to commercial NPs, achieved *via* controlled nanoprecipitation in a high ionic strength medium. This property facilitated detection under high circulation rates and significant background autofluorescence. These NPs allowed for the detailed monitoring of NP pharmacokinetics and biodistribution in the cerebral vasculature of live mice, including real-time tracking of NP extravasation across the blood–brain barrier (BBB) through the implanted cranial window.

**Table 1:** Summary of studies involving IVM for the investigation of NPs *in vivo*, including the NP type, observation route in the body, fluorescence property enabling NP detection, and the observed phenomenon

Nanoparticle type	Observation route	Fluorescence property	Observed phenomenon	Ref.
PF-68-PLGA (polymeric)	Cranial window	Lumogen-red loading	NP circulation in brain vasculature	24
PMMA-SO_3_H (polymeric)	Cranial window	Octadecyl rhodamine B	Circulation through meningeal vessels	25
FL (lipidic)	Extracorporeal placement of tumor	DiD + DiO	Extravasation through micro- and macro-leakages	26
MSNs (inorganic)	Abdominal window	Fluorescein isothiocyanate (FITC)	Dependance of surface charge on liver uptake	32
Au–SiO_2_ (inorganic)	Ear vasculature	Intrinsic gold multiphoton luminescence	Circulation	35
SNPs-PEG-RGD-FITC (inorganic)	Cranial window	Fluorescein isothiocyanate (FITC)	Penetration and distribution in glioma	79
CLIO-AF647 (inorganic)	Surgical exposure of skull	Alexa fluor 647 conjugation	NP uptake in calvaria	80
cRGD formulations (lipidic)	Extracorporeal placement of tumor	ATTO633-PE and rhodamine-PE	Effect of peptide conjugation on tumor targeting	81
C'dots (inorganic)	Metatarsal isolation surgery	Cy5 dye encapsulation	Sex-specific osteocyte metabolism	82
Gd-based AGuIX	Extracorporeal placement of kidney	Rhodamine B labeling	Kidney clearance	83
FMNs (inorganic)	Dorsal window	Cy5.5 doping of silicas shell	NP tumor delivery with magnetic targeting	84
BPBBT-HSA (organic)	Extracorporeal placement of tumor	BPBBT conjugation	Delivery mechanism to tumor	86
Ferumoxytol (inorganic)	Cranial window	FITC conjugation	Multiscale imaging of NP delivery to glioblastoma	91
α-Melittin-NPs	Abdominal window	DiR-BOA labeling	Targeting of liver sinusoidal endothelial cells	94
Nanobubbles	Dorsal window	Porphyrin/Texas red	Ultrasound-triggered extravasation	88
RGS-SWNTs (inorganic)	Dorsal window	Cy5.5 conjugation	Glioblastoma NP targeted delivery	90
FedEcs (lipidic)	Cranial window	F888/Dil	Post-stroke NP accumulation	92

Zhang *et al.* investigated the use of silicon NPs (SNPs-PEG-RGD-FITC) in a syngeneic orthotopic glioma mouse model to assess their tumor-targeting abilities with IVM.^79^ A chronic cranial window was implanted to enable real-time, high-resolution imaging of the brain tumor. The main findings indicated that arginine–glycine–aspartate peptide- (RGD-) conjugated NPs exhibited enhanced infiltration and binding within the glioma tissue, as evidenced by the conjugated fluorophore (FITC). These NPs penetrated and distributed through the tumor, showing reduced wash-out and sustained retention, in contrast to control NPs without RGD, which accumulated less effectively.

**Fig. 6:**
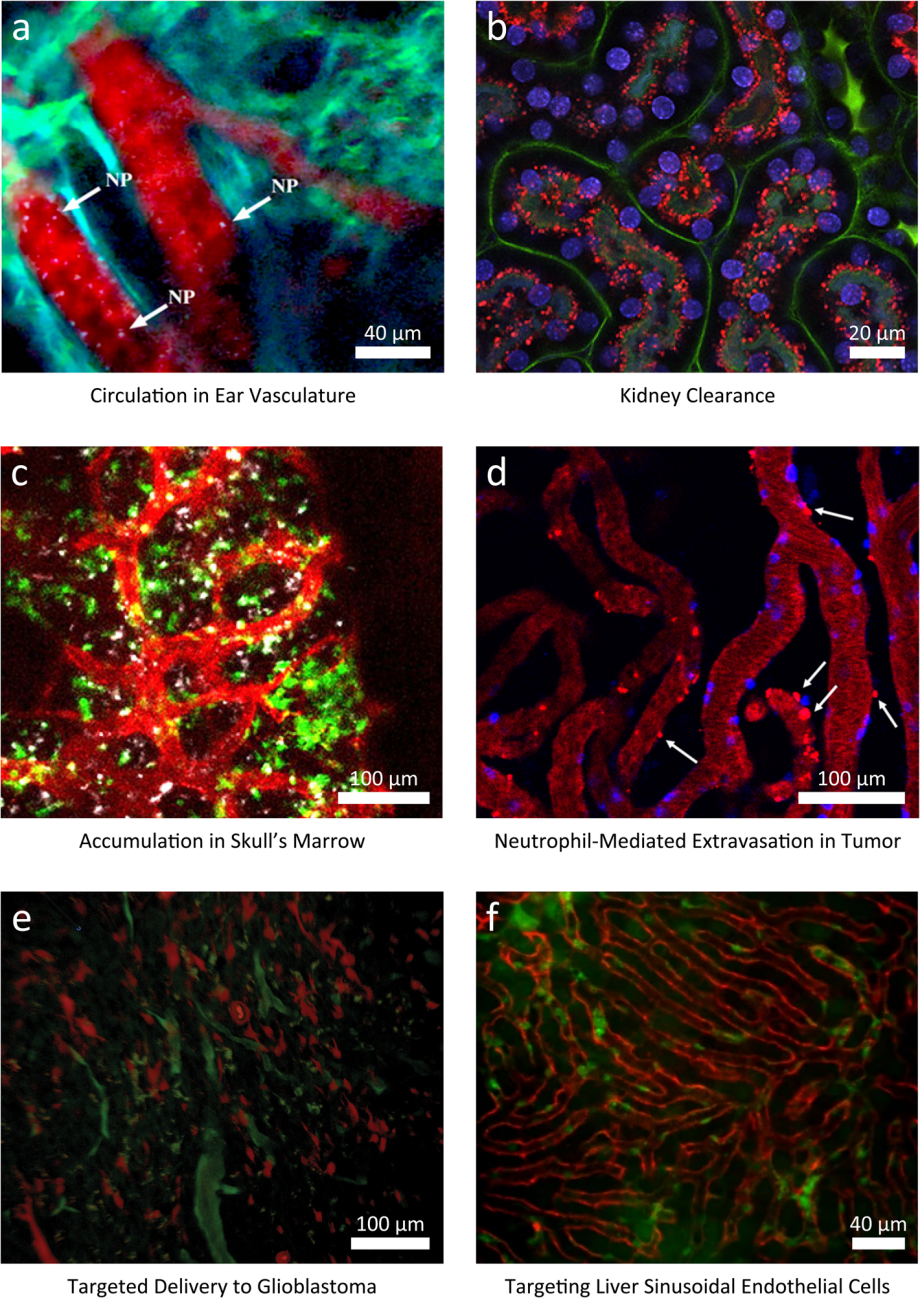
Representative IVM images investigating nanoparticle- (NP-) cell interactions. (a) Label-free tracking of gold–silica NPs (white) circulating in ear vasculature (background vessel autofluorescence, red). *λ*_ex_ = 920, 1040 nm; *P* = 1.3 mW. Adapted from Burkitt *et al.* (CC-BY 4.0).^35^ Scale bar, 40 µm. (b) Renal elimination of Rhodamine B-labeled (red) gadolinium-based NPs (AGuIX) in real time 4 h after intravenous injection (*λ*_ex_ = 800 nm; *P* = 60 mW). Staining of the nucleus (Hoechst 33342, blue) and vessels (FITC-Dextran, green). Adapted with permission from Sancey *et al.*^83^ Scale bar, 20 µm. (c) NP accumulation in the calvaria after intravenous injection of cross-linked iron oxide- (CLIO-)AF647 (white, *λ*_ex_ = 633 nm). Staining of monocytes (Cx3cr1GFP/+, green, *λ*_ex_ = 488 nm), vessels (CD31+, red, *λ*_ex_ = 561 nm). Adapted from Tiwari *et al.* (CC-BY 4.0).^80^ Scale bar, 100 µm. (d) Liposome-DiD (red) extravasation 20 min after intravenous administration to 4T1 tumor-bearing mice, with microleakage formation (white arrows) in tumor. Staining of the neutrophils (Ly6G^+^, blue). *λ*_ex_ = 405, 488, 561, and 647 nm. Adapted from Garanina *et al.* (CC-BY 4.0).^87^ Scale bar, 100 µm. (e) Ferumoxytol NPs conjugated with a matrix metalloproteinase-14-cleavable FITC-conjugated (green) vascular disrupting agent for targeted delivery to glioblastoma (red fluorescent protein- (RFP-) labeled C6 tumor cells). *λ*_ex_ = 920 nm. Adapted from Saladino *et al.* (CC-BY 3.0).^91^ Scale bar, 100 µm. (f) α-Melittin NPs (DiR-BOA, red) targeting the liver sinusoidal endothelial cells (LSECs) of Actb-EGFP mice (cells in green) after intravenous injection. Adapted from Yu *et al.* (CC-BY 4.0).^94^ Scale bar, 40 µm.

Gold–silica nanoparticles (Au–SiO_2_ NPs) were employed to study their circulation through the ear vasculature of mice without the need for a window implantation.^35^ The investigation utilized IVM to observe the accumulation and flow of these nanoparticles in real-time. Au–SiO_2_ NPs were selected for their intrinsic multiphoton luminescent properties, which allowed for clear visualization without additional labelling. The main findings highlighted that these NPs could be effectively tracked and quantified within the blood vessels as a function of time (Fig. 6a).

In another study, IVM was employed to investigate the clearance of gadolinium-based NPs (AGuIX) synthesized as a simultaneous MRI contrast agent and radiosensitizer: through the extracorporeal placement of the kidney, the NP renal elimination was followed with IVM in real time, highlighting that AGuIX NPs were mostly cleared within 1 week. Furthermore, the NP kinetics in the kidney observed with IVM were correlated with ICP-OES and LIBS, with consistent results. IVM enabled estimation of the time of highest AGuIX uptake, recorded at 4 h after NP administration (Fig. 6b).^83^

The uptake dynamics of cross-linked iron oxide (CLIO) NPs in hematopoietic bone marrow was recently assessed with a cross-modal imaging approach including IVM, highlighting a significant slowdown of CLIO-AF647 NPs in the vascular niches of the inflamed bone marrow and evidenced the calvaria's role as a distribution center for NP transport (Fig. 6c).^80^ The tumor NP delivery of fluorescent magnetic NPs (FMNs) with magnetic targeting was assessed with an ectopic xenograft model of EGFP-transfected U87MG human glioblastoma. IVM observation of the tumor was enabled by the surgical implantation of a dorsal skinfold chamber.^84^ IVM demonstrated the efficacy of magnetic targeting of FMNs in a native state and *in vivo*.

Khalin *et al.* investigated the *in vivo* visualization of Lumogen red-loaded poly(lactic-*co*-glycolic acid) (PLGA) NPs coated with poloxamer 188 (Pluronic F-68, PF-68), revealing that the PF-68 coating increased their circulation time within the brain vasculature compared to bare NPs. After systemic injection, the coated NPs exhibited detectable fluorescence intensity for over an hour, while the bare NPs rapidly diminished within minutes, indicating that PF-68-coated NPs are circulating longer, making them more promising for biomedical applications.^24^

In a study utilizing IVM on extracorporeally placed 66cl4 tumors and on healthy ear tissues, Sofias *et al.* investigated the difference in tumor uptake and blood circulation of co-injected α_v_β_3_-integrin-specific cyclic arginine–glycine–aspartate peptide- (cRGD-) NPs (treatment) and cyclic arginine–alanine–aspartate peptide- (cRAD-) NPs (non-specific control peptide). The shorter blood circulation half-lives of cRGD-NPs, coupled with increased uptake in the liver and spleen compared to cRAD-decorated analogues, reduced their effectiveness in targeting tumors.^81^ IVM highlighted that cRGD decoration could actually reduce the amount of therapeutic agent delivered to the tumor and potentially lead to increased adverse side effects. Overall, this study evidenced the importance of focused studies on NP pharmacokinetics *in vivo* with IVM.

A research group introduced IVM to dynamically track the hepatic metabolism of NPs with subcellular resolution in real time using a hepatic imaging window. Using mesoporous silica nanoparticles (MSNs), the study found that positively charged MSNs are significantly uptaken by hepatocytes, while negatively charged MSNs are rapidly taken up by Kupffer cells.^32^ This differential uptake impacts hepatotoxicity and clearance pathways, showcasing IVM's potential in the preclinical evaluation of NPs with varied surface modifications. Naumenko *et al.* employed IVM to observe the *in vivo* behavior of fluorescent (DiD/DiO)-labeled liposomes (FL) within tumor xenografts. This approach enabled the dynamic tracking of liposome extravasation with spatial and temporal resolution, evidencing two distinct leakage patterns: microleakages, facilitated by neutrophils that temporarily opened the vascular barrier (found both in malignant and healthy tissues), and less stable, dynamic macroleakages localized predominantly on the tumor–host interface. The frequency of micro- and macroleakages was higher in proximity of neutrophils, suggesting a neutrophil-assisted extravasation.^26^

Cornell Prime Dots (C'Dots) have recently been utilized to facilitate rapid visualization of the osteocyte dendritic network and intracellular dynamics with IVM, enabled by a metatarsal isolation surgery. Notably, the study found variations in C'Dot uptake and retention between male and female mice, with significant implications for understanding sex-specific osteocyte metabolism and bone health, particularly in the context of integrin function and trafficking.^82^ In another study, the binding of RGD quantum dots conjugates to tumor luminal endothelium was studied with IVM, demonstrating the ability to directly follow the specific binding of NPs to biomolecules expressed on tumor (binding rate per field-of-view).^85^ Albumin-bound NPs were tracked with IVM through a lipophilic NIR-II fluorescence molecule, BPBBT, which exhibits high binding affinity to human serum albumin (HSA). By testing several formulations of BPBBT-HSA NPs, the authors demonstrated that endothelial transcytosis was the dominant pathway for the delivery of albumin-bound NPs into the tumor parenchyma.^86^

The delivery of liposomes, poly(lactic-*co*-glycolic acid) (PLGA) NPs, and magnetite NPs to 4T1 mammary adenocarcinoma was investigated with IVM, to assess the contribution of neutrophils to NP tumor targeting: the results highlighted that the NP type largely determined the extent of the neutrophil-mediated delivery to the tumor (Fig. 6d).^87^ Pellow *et al.* employed IVM to investigate the ultrasound-triggered nanobubble generation and extravasation in the tumor vasculature, using a dorsal window chamber.^88^ Extravascular fluorescence in treated mice increased five folds compared to its initial intensity 15 min after administration.

Miller *et al.* investigated the pharmacokinetics and pharmacodynamics of a fluorescent platinum(iv) pro-drug bound to a polymer platform (PLGA-*b*-PEG) resulting in therapeutic NPs. The NP delivery to a xenografted tumor model was tracked with time-lapse IVM using a dorsal window chamber. These studies highlighted that the prodrug encapsulation enabled a longer circulating half-life and influenced the spatial distribution within the tumor with NPs primarily accumulating in perivascular cells.^89^

Smith *et al.* studied the delivery of single-walled carbon nanotubes (SWNTs) functionalized with the RGD peptide (RGD-SWNTs) to xenografted human glioblastoma tumor cells with a dorsal skinfold chamber. IVM images revealed a time-dependent effect of the active targeting, limited to early-stage vascular binding and late-stage tumor cell binding.^90^

Furthermore, IVM was recently employed to assess the delivery of ferumoxytol-based theranostic NPs to glioblastoma, developing a methodology for *in vivo* multiscale imaging using IVM (cellular level) and MRI (macroscopic level) (Fig. 6e).^91^ Khalin *et al.* developed fluorescent lipid nanodroplets carrying a FRET-based cargo delivery system (FedEcs). With IVM, the authors assessed the *in vivo* stability of LNDs and showcased their preferential accumulation in microvascular clots, as well as cargo delivery to the brain parenchyma following cerebral ischemia.^92^

In the study by Li *et al.*, IVM provided real-time visualization of cellular and molecular dynamics to monitor the biodistribution and localization of glutathione-protected gold nanoclusters (AuNCs) conjugated with a photosensitizer (rose bengal) and RGD to target and treat intracranial glioma tumors with low-dose X-ray photodynamic therapy. The authors assessed the integrity of the blood–brain barrier and evaluated the impact on normal brain tissue and on the tumor. These findings supported the conclusion that low-dose X-PDT could be an effective clinical treatment for high-grade gliomas.^93^

The ability to target liver sinusoidal endothelia cells with α-melittin NPs has been investigated with IVM, demonstrating that they blocks liver metastasis formation and prolongs the survival rates of mice (Fig. 6f).^94^

IVM has also been employed in preclinical research with other species, besides mice. A recent study investigated the differential sequestration of NPs by macrophages and scavenger endothelial cells using IVM. They examined Pacific Blue-labeled silica (SiO_2_) NPs (70 nm in diameter) in zebrafish embryos, visualizing their accumulation, intracellular localization, and sequestration kinetics *in vivo*.^95^ The main findings revealed distinct accumulation in the caudal vein plexus and sequestration behaviors by macrophages and endothelial cells, with macrophages rapidly ingesting NPs *via* macropinocytosis and scavenger endothelial cells (SECs) gradually uptaking NPs through scavenger receptors into endolysosomal compartments. Overall, these findings demonstrated the importance of IVM in distinguishing different extravasation mechanisms and the interactions between NPs and the tissue microenvironment.

## Intravital imaging and quantitative analysis

**Fig. 7:**
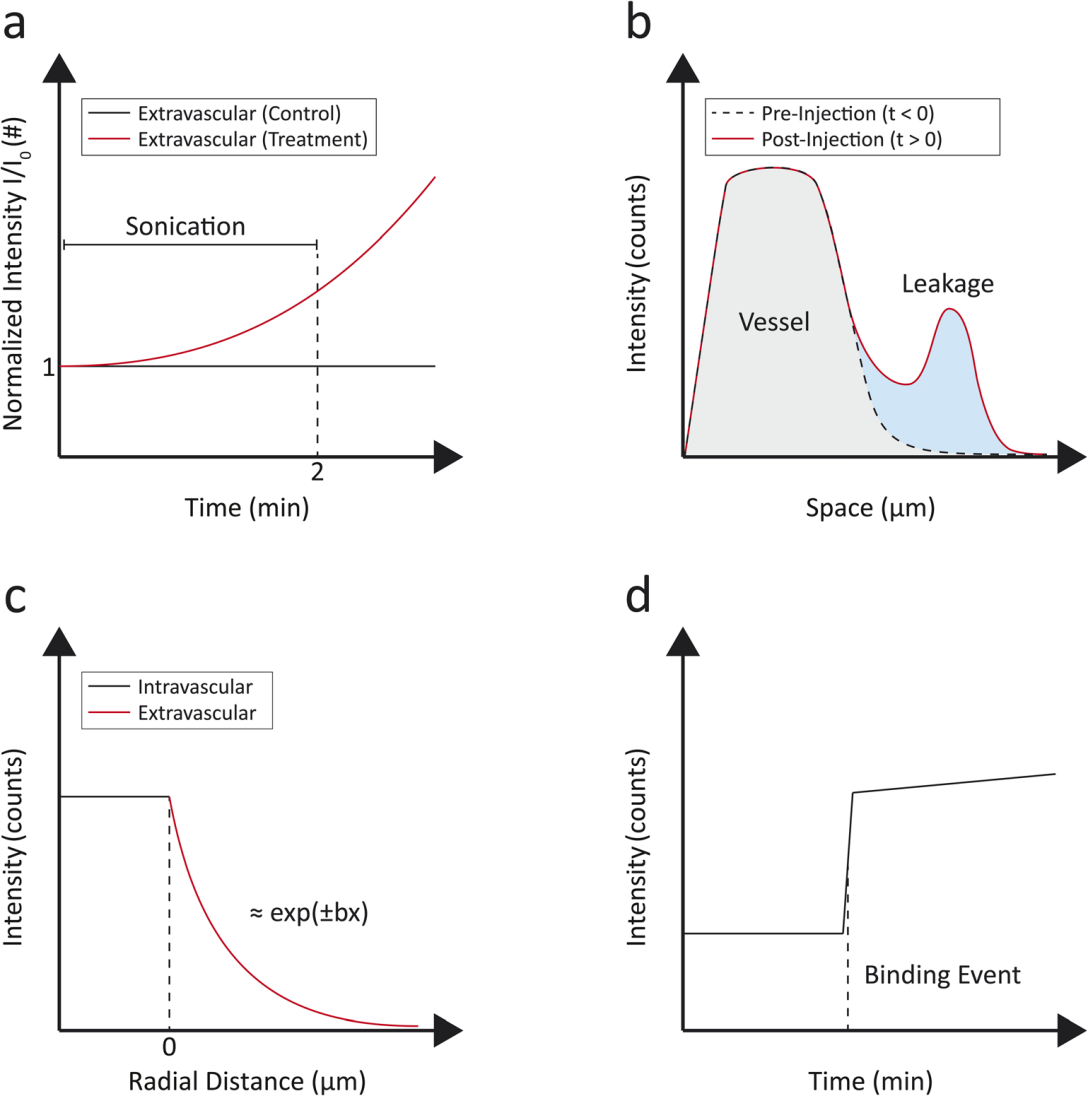
Representative schematic plots for IVM analysis. (a) Visual monitoring of ultrasound-induced extravasation from tumor vasculature:^88^ extravascular compartment fluorescence (normalized at *t* = 0, and per surface area) recorded during and after sonication in a selected imaging plane in treated mice (with ultrasound, in red) and control mice (without ultrasound, in black). (b) Liposomes extravasation tracking in tumor with evolution of leakages before and after appearance.^26^ Leakages (in blue) are localized within few microns from the vessel (in grey). (c) Extravasation of ferumoxytol-based ultrasmall superparamagnetic iron oxide (USPIO) nanoparticles from brain tumor (glioblastoma) vasculature (black). Extravascular signal (red) is fitted with a decaying exponential curve and the fluorescence spatial decay rate is recorded.^91^ (d) Binding mechanism of cRGD-nanoemulsion-positive immune cell to tumor vasculature. The recorded fluorescence intensity as a function of time from selected regions of interest highlights stepwise cell binding events.^81^

Quantitative analysis *via* IVM is critically important yet still underexplored in NP interaction studies at the cellular level *in vivo*, primarily due to technical challenges, the complexity of biological systems, and the limited availability of this imaging technology. IVM, with its ability to acquire high-resolution real-time multiplexed imaging of cellular and subcellular processes, can provide essential insights into the NP pharmacokinetics and pharmacodynamics, including distribution, cellular uptake, endocytosis, and intracellular trafficking within native physiological contexts. Integrating quantitative assessments into IVM can enable a deeper understanding of NP interactions, receptor-ligand dynamics, bioavailability, and signaling pathways. This approach is fundamental for the rational design and optimization of nanotherapeutics, improving targeting specificity, efficacy, and biosafety. Some examples of IVM quantifications were schematically depicted in Fig. 7. Pellow *et al.* demonstrated the real-time quantification of ultrasound-triggered vascular permeability using an integrated acoustic and microscopy setup. The study compared two groups: a treatment group subjected to ultrasound and a control group without ultrasound exposure. During a 2-minute sonication, extravascular fluorescence intensity (normalized per volume and initial intensity) in the treatment group doubled, while control group levels remained constant, as schematically shown Fig. 7a. Over a 65-minute span, the treated mice exhibited an increase in extravascular fluorescence to five times the initial intensity within 15 minutes, whereas the control group showed only a modest rise to 1.3 times, due to passive targeting. Additionally, intravascular fluorescence increased initially post-injection in both groups but gradually declined over the hour.^88^ The quantification of these IVM observations specifically underscored the enhanced vascular permeability and NP distribution resulting from ultrasound treatment. Another quantitative approach was presented by Naumenko *et al.*, who characterized two distinct patterns of liposome extravasation in tumors using IVM. The more frequently observed pattern, termed microleakage, involved localized leakage into the perivascular area, limited to within 20 µm from the blood vessels. This type of extravasation resulted in fluorescence intensity being evenly distributed within the microleakage region, surpassing the intensity within the vessel lumen, and stabilizing rapidly within minutes without further changes over time. Conversely, the less common macroleakage pattern encompassed a larger interstitial area, penetrating deeper into tissues, and exhibited a diffusion gradient with dynamic changes in fluorescence intensity that remained lower than the intensity of circulating liposomes. Macroleakages demonstrated repeated waves of fluorescence extravasation from the same leakage spot, reflecting NP diffusion in the interstitial space. The integrity of liposome–dye complexes was confirmed using liposomes labeled with different dyes and dual-labeled liposomes, ensuring the observed extravasation patterns were consistent across various formulations.^26^ This example of IVM quantification was schematically depicted in Fig. 7b. The ability to distinguish between micro- and macroleakage patterns with IVM can help the understanding on how different NP types behave in physiological environments, guiding the design and optimization of targeted therapies.

Saladino *et al.* conducted an analysis of NP extravasation using IVM and MRI at extended times post-injection (24 hours). They assessed the fluorescence spatial decay rate of signal intensity profiles across blood vessels to compare two NP formulations, one with a vascular disrupting agent and one without. The results revealed that the formulation containing the vascular disrupting agent had a lower decay rate (0.8 ± 0.4 µm^−1^), indicating enhanced diffusion away from the blood vessels compared to the formulation without the agent (1.3 ± 0.6 µm^−1^). Additionally, the quantification highlighted a strong positive correlation between tumor *T*_2_-relaxation time (measured by MRI) and the spatial decay rate (measured by IVM), demonstrating a consistent relationship between vascular permeability and NP distribution.^91^ The fluorescence spatial decay rate was estimated by applying a decaying exponential fit, as schematically shown in Fig. 7c.

By allowing for the detailed assessment of vascular permeability and NP diffusion, IVM can guide the optimization of NP formulations, improving their targeting efficacy and safety.

Another analytical approach was pursued by Marios Sofias *et al.*, who reported differences in the tumor delivery behavior of cRGD NPs compared to their control analogues. Within minutes after administration, a sudden uptake of cRGD-NPs by circulating immune cells was observed and confirmed through intravital CD45 staining. This uptake was quantitatively assessed over time, showing extensive cellular internalization of cRGD-NPs. In contrast, cRAD-NPs exhibited negligible leukocyte uptake during this period. IVM allowed for quantification of the binding events of cRGD-NP positive cells to tumor vasculature. The authors observed that binding events occurred abruptly and targeted entire clusters rather than gradually accumulating individual NPs (Fig. 7d). IVM highlighted this stepwise binding, thus demonstrating the mechanism of targeting and adhesion of immune cells loaded with cRGD-NPs to the tumor vasculature.^81^ Overall, these examples highlight the importance of performing quantitative IVM analyses, which enhance our understanding of NP behavior *in vivo*, thus promoting the advancement of nanomedicine from experimental research to impactful clinical applications.

## Reporting checklist for intravital microscopy studies in nanomedicine

A standardized minimum information checklist for IVM of NPs would greatly enhance reproducibility and cross-study comparisons. Research articles should include animal information (strain, sex, age, and number of animals used), details of window surgery, perioperative care, and the post-surgical waiting periods to ensure tissue recovery. The anesthesia regimen and measures for temperature and physiological control during imaging should be specified. NP characterization must cover surface chemistry, size distribution, surface modifications, loading, and photophysical properties, with explicit inclusion of protein corona characterization under relevant biological conditions. The dosing strategy (route, concentration, injection volume, and timing) should be provided alongside a full description of the IVM setup (excitation wavelengths, objective NA, laser power, detection). A laser power that does not cause cell stress and/or photodamage at a given magnification and scan angle should be used. Future IVM studies in nanomedicine should include quantitative kinetic analyses, such as Patlak permeability modeling and mean squared displacement (MSD)-based diffusion models.^96–98^ Rigorous statistical analysis should be applied, including mixed-effects models for longitudinal datasets and appropriate multiple-comparison controls. To ensure data integrity, calibration and quality control procedures (including power measurements, resolution checks and fluorophore stability) should be stated, and criteria for blinding, randomization, and data exclusions clearly defined. Finally, data and code availability should be specified to allow independent validation and reuse. Together, such a checklist would set a transparent standard, reduce variability, and promote more rigorous IVM studies.

## Summary and outlook

IVM has emerged as a transformative tool in nanomedicine, providing novel insights into the dynamic interactions between NPs and the cellular environment at high spatial and temporal resolution. This review highlighted how IVM has advanced our understanding of key processes such as NP biodistribution, extravasation, cellular uptake, and clearance within physiologically relevant microenvironments. By enabling real-time visualization of NP behavior in the native tissue and vasculature context, IVM bridges the gap between *in vitro* and *in vivo* studies, offering critical validation for preclinical models and targeting strategies. In fact, while many *in vivo* techniques (MRI, PET, IVIS) allow macroscopic observation of NP biodistribution, IVM uniquely provides complementary microscopic insights. IVM is most critical when cellular-resolution dynamics, including NP interactions with endothelial and other stromal cells, or vascular behavior influence interpretation, whereas *ex vivo* or mesoscopic approaches suffice for whole-organ distribution or bulk pharmacokinetics. Despite significant progress, several challenges remain: the current IVM approaches are often limited by imaging depth and field-of-view constraints. For dynamic studies, multiphoton imaging is limited by its small field of view, requiring repeated imaging of the same area to track cellular events over time. Furthermore, the need for surgical windows makes the experimental setting complex. Moreover, quantitative interpretation of IVM data still requires standardized metrics and integration with complementary techniques such as advanced computational modeling. In the future, IVM-based research should focus on investigating deeper tissues and organoids. The development of more sensitive and multiplexed nanoprobes will enable simultaneous tracking of multiple NP formulations and cell populations. In the future, expanding IVM studies beyond mice to models such as rats and minipigs will enable investigation of immune interactions and NP pharmacokinetics in systems with greater physiological and immunological relevance to humans. As the field evolves, IVM is very promising for the rational design of nanomedicines, the identification of new biological interactions and key-factors to enhance nanoparticle delivery. Finally, IVM can enable personalized nanotherapeutic design by using patient-derived tumors to characterize microenvironmental features that influence NP targeting; integrating IVM into the drug development pipeline could accelerate the clinical translation of nanomedicines.

## Conflicts of interest

There are no conflicts to declare.

## Data Availability

No primary research results, software or code have been included, and no new data were generated as part of this review.
